# Association of Smartwatch-Based Heart Rate and Physical Activity With Cardiorespiratory Fitness Measures in the Community: Cohort Study

**DOI:** 10.2196/56676

**Published:** 2024-06-13

**Authors:** Yuankai Zhang, Xuzhi Wang, Chathurangi H Pathiravasan, Nicole L Spartano, Honghuang Lin, Belinda Borrelli, Emelia J Benjamin, David D McManus, Martin G Larson, Ramachandran S Vasan, Ravi V Shah, Gregory D Lewis, Chunyu Liu, Joanne M Murabito, Matthew Nayor

**Affiliations:** 1 Department of Biostatistics School of Public Health Boston University Boston, MA United States; 2 Section of Endocrinology, Diabetes, Nutrition, and Weight Management, Department of Medicine Boston University Chobanian & Avedisian School of Medicine Boston, MA United States; 3 Department of Medicine University of Massachusetts Chan Medical School Worcester, MA United States; 4 Center for Behavioral Science Research, Department of Health Policy & Health Services Research Boston University, Henry M. Goldman School of Dental Medicine Boston, MA United States; 5 Boston University's and National Heart, Lung, and Blood Institute's Framingham Heart Study Framingham, MA United States; 6 Section of Preventive Medicine and Epidemiology and Cardiovascular Medicine Departments of Medicine and Epidemiology Boston University Chobanian & Avedisian School of Medicine and School of Public Health Boston, MA United States; 7 Cardiology Division Department of Medicine University of Massachusetts Chan Medical School Worcester, MA United States; 8 Department of Quantitative Health Sciences University of Massachusetts Chan Medical School Worcester, MA United States; 9 Cardiology Division Vanderbilt Translational and Clinical Research Center Vanderbilt University Medical Center Nashville, TN United States; 10 Cardiology Division Department of Medicine Massachusetts General Hospital, Harvard Medical School Boston, MA United States; 11 Pulmonary Critical Care Unit, Department of Medicine Massachusetts General Hospital Boston, MA United States; 12 Section of General Internal Medicine Department of Medicine Boston University Chobanian & Avedisian School of Medicine Boston, MA United States; 13 Sections of Cardiology and Preventive Medicine and Epidemiology Department of Medicine Boston University Chobanian & Avedisian School of Medicine Boston, MA United States

**Keywords:** mobile health, smartwatch, heart rate, physical activity, cardiorespiratory fitness, cardiopulmonary exercise testing

## Abstract

**Background:**

Resting heart rate (HR) and routine physical activity are associated with cardiorespiratory fitness levels. Commercial smartwatches permit remote HR monitoring and step count recording in real-world settings over long periods of time, but the relationship between smartwatch-measured HR and daily steps to cardiorespiratory fitness remains incompletely characterized in the community.

**Objective:**

This study aimed to examine the association of nonactive HR and daily steps measured by a smartwatch with a multidimensional fitness assessment via cardiopulmonary exercise testing (CPET) among participants in the electronic Framingham Heart Study.

**Methods:**

Electronic Framingham Heart Study participants were enrolled in a research examination (2016-2019) and provided with a study smartwatch that collected longitudinal HR and physical activity data for up to 3 years. At the same examination, the participants underwent CPET on a cycle ergometer. Multivariable linear models were used to test the association of CPET indices with nonactive HR and daily steps from the smartwatch.

**Results:**

We included 662 participants (mean age 53, SD 9 years; n=391, 59% women, n=599, 91% White; mean nonactive HR 73, SD 6 beats per minute) with a median of 1836 (IQR 889-3559) HR records and a median of 128 (IQR 65-227) watch-wearing days for each individual. In multivariable-adjusted models, lower nonactive HR and higher daily steps were associated with higher peak oxygen uptake (VO_2_), % predicted peak VO_2_, and VO_2_ at the ventilatory anaerobic threshold, with false discovery rate (FDR)–adjusted *P* values <.001 for all. Reductions of 2.4 beats per minute in nonactive HR, or increases of nearly 1000 daily steps, corresponded to a 1.3 mL/kg/min higher peak VO_2_. In addition, ventilatory efficiency (V_E_/VCO_2_; FDR-adjusted *P*=.009), % predicted maximum HR (FDR-adjusted *P*<.001), and systolic blood pressure-to-workload slope (FDR-adjusted *P*=.01) were associated with nonactive HR but not associated with daily steps.

**Conclusions:**

Our findings suggest that smartwatch-based assessments are associated with a broad array of cardiorespiratory fitness responses in the community, including measures of global fitness (peak VO_2_), ventilatory efficiency, and blood pressure response to exercise. Metrics captured by wearable devices offer a valuable opportunity to use extensive data on health factors and behaviors to provide a window into individual cardiovascular fitness levels.

## Introduction

Impaired cardiorespiratory fitness (CRF) is a powerful predictor of health outcomes including incident cardiovascular disease (CVD) events and mortality [1-6]. To optimize the prevention of CVD, the American Heart Association supports the clinical assessment of CRF as a “vital sign” in all adults [6]. While routine CRF assessment may be a valuable tool, given the possibility of modifying CRF with greater physical activity and other lifestyle modifications [7,8], frequent CRF assessment at the population level is not currently feasible, especially in resource-limited settings. Additional markers that track CRF are therefore desirable to gauge general CRF and changes with lifestyle modifications or therapeutic interventions.

Metrics derived from wearable devices present a unique opportunity to leverage large amounts of collected data on health factors and behaviors to provide a window into individual CRF levels. With the rise in technology use [9], nearly 70% of US adults report tracking at least 1 health measure [10,11], including millions of individuals who track their steps using wearable devices [12]. In addition to daily steps taken, wearable devices permit continuous monitoring of heart rate (HR) in real-world environments over long periods of time. The American Heart Association recommends that individuals get to know their HR to help self-monitor CRF [13]. Resting HR associated with CRF [14] and lower HR have also been associated with a lower risk for mortality and adverse cardiovascular outcomes [15,16]. In addition, routine physical activity is known to be associated with improvements in CRF in women and men and across cardiovascular risk [7]. However, the relationships between HR and physical activity indices from consumer-grade wearables to gold standard assessments of CRF measures from cardiopulmonary exercise testing (CPET) are sparse.

We investigated the association between the measures of CRF obtained from CPET with smartwatch measures of physical activity and nonactive HR in a community-based sample of middle-aged and older adults from the electronic Framingham Heart Study (eFHS). We investigated both peak VO_2_ and novel CPET measures that capture complementary dimensions of CRF. We hypothesized that higher levels of CRF (higher peak VO_2_) are associated with lower average HR and greater steps per day over follow-up. The ability of individuals to track real-world physiologic measures that reflect overall CRF in real time may encourage engagement with lifestyle behaviors that result in improvements in not only cardiovascular health but also overall health.

## Methods

### Study Sample

**Figure 1:**
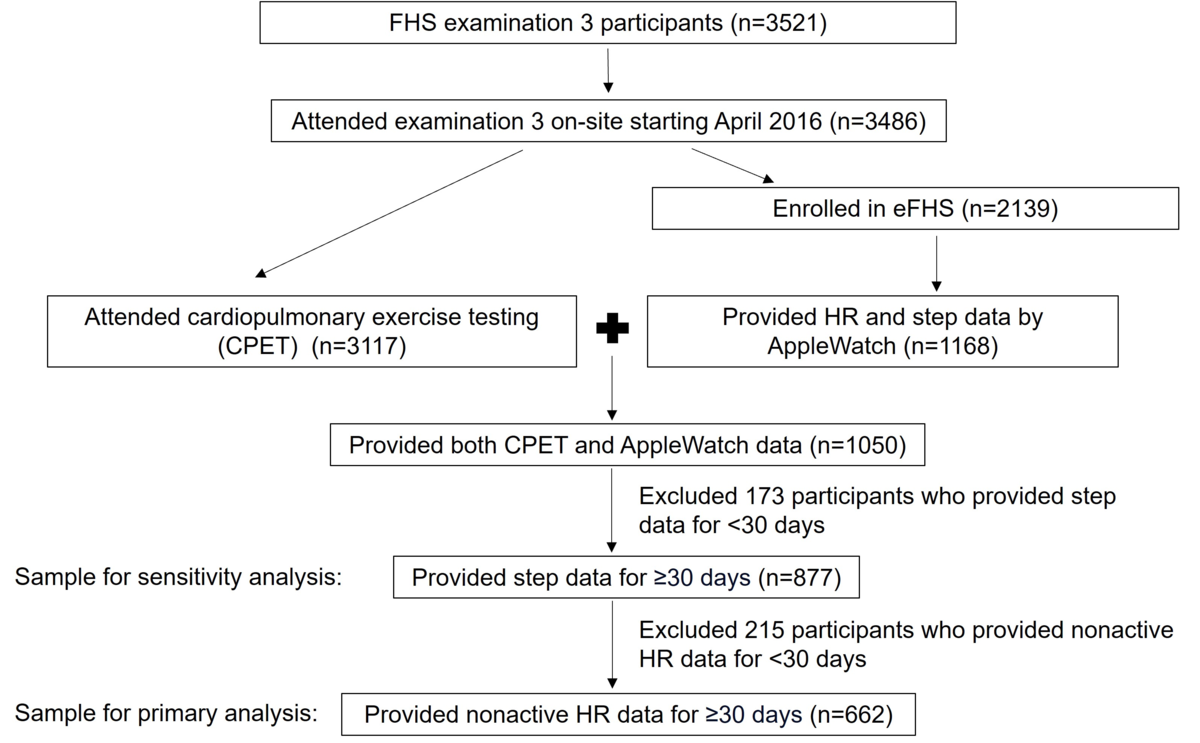
Flowchart of analytic sample inclusion. eFHS: electronic Framingham Heart Study; FHS: Framingham Heart Study; HR: heart rate.

The Framingham Heart Study (FHS) is a community-based, prospective study initiated in 1948 in the town of Framingham, Massachusetts, to study CVD and its determinants [17-19]. In 2002-2005, the grandchildren of the original participants, the Third Generation cohort (Gen 3), a cohort of multiple ancestries (Omni 2), and a cohort of New Offspring Spouses were enrolled and underwent examinations every 6-8 years [17]. At the time of research examination 3 (2016-2019; n=3521), the participants were invited to the eFHS. The eFHS enrolled 2139 English-speaking FHS participants who owned a smartphone (iPhone 4S or newer iPhones with at least iOS 8.2 or Android Phones) and provided consent. Participants with iPhones were offered a study smartwatch (Apple Watch Series 0) to record step count and HR data for 1 year (or more if they desired) following enrollment. The participants who owned an Apple Watch were allowed to use their own smartwatch. The participants in eFHS were instructed to wear the smartwatch after waking up in the morning and remove the watch at bedtime for charging. The eFHS included a smartphone app for electronic consent, health questionnaires, notifications, and integration with the smartwatch. The eFHS research technician assisted participants with app download and smartwatch setup and provided participants with a written protocol for remote use that included information on how to download the app, enter registration information, sign the consent forms, enable notifications on the smartphone, and set up the smartwatch. In research examination 3, the participants were also offered participation in CPET [20]. Of the 3521 participants attending the examination, 3117 completed the CPET, of whom 1050 enrolled in eFHS with an iPhone and chose to use a smartwatch (Figure 1). We further excluded 388 participants who did not contribute 30 or more days of HR or step data leaving a final sample for our primary analysis of 662 participants.

### Ethical Considerations

The Boston University Medical Campus institutional review board reviewed and approved the study (H-36586 and H-32132). All participants provided informed consent for their participation in eFHS and for participation in examination 3, which included the CPET. Data were deidentified for analysis. Participants were not compensated for participating in the study.

### CPET Measures (Independent Variables)

The CPET protocol used in FHS has been described previously [7,21,22] and is briefly summarized through the subsequent procedures. Maximal effort CPET was conducted on a cycle ergometer (Lode) with breath-by-breath gas exchange data measured (Medgraphics). The protocol consisted of 3 minutes of unloaded exercise and incremental ramp exercise using 1 of 2 ramp protocols (15 and 25 W/min). For this study, the primary CPET variables of interest were the “gold standard” measures of CRF: peak oxygen uptake (VO_2_; in mL/kg/min), which was measured as the highest 30-second median value during the final minute of exercise and % predicted peak VO_2_ (calculated using the Wasserman-Hansen equations) [23]. The VO_2_ was also measured at the ventilatory anaerobic threshold (VAT), which was assessed using the V-slope method [24]. We evaluated ventilatory responses to exercise via ventilatory efficiency (assessed by the ratio of minute ventilation to carbon dioxide production [V_E_/VCO_2_] and calculated as the lowest 30-second median value observed during exercise) [21], HR responses to exercise (assessed by the % predicted maximum HR achieved, with the predicted maximum HR calculated using the Tanaka formula) [25], and blood pressure/vascular responses to exercise (assessed by systolic blood pressure-to-workload slope [SBP/W slope] and calculated as [peak SBP–rest SBP]/peak workload) [26].

### Smartwatch Measures (Dependent Variables)

In this study, participants’ HR and step counts were collected from smartwatches for up to 3 years, with a median follow-up of 128 (IQR 65-227) days.

The Apple Watch has built-in sensors including an accelerometer, gyroscope, and optical heart sensor to record the user’s HR and determines the motion context, that is, whether the user is in sedentary or active status [27]. The Apple Watch Series 0 captured incomplete information on motion context, with only 31% of HR measures associated with information on motion context. Therefore, we developed a classification algorithm to infer the sedentary state for HR measures without motion context information. The smartwatch typically returns HR data every few minutes, and more frequent HR measures are returned when users are active. Using the HR data with known motion context, we observed that the recording intervals were less than 1 minute for ~99% of HR measures with active status (Figure S1 in Multimedia Appendix 1). On the other hand, ~99% of HR measures with sedentary status had a recording interval greater than 1 minute. Based on the observed HR data with known motion context status, we inferred the motion context status for HR data with unknown status. We defined an HR measure to be “nonactive” if the HR measure satisfied the following conditions: (1) the recording interval between the adjacent HR measures was greater than 1 minute and (2) hourly step counts were less than 30 steps within the same hour when the HR was recorded. After inferring the motion context status for the 69% of HR measures that lacked motion context information directly returned from Apple Watch Series 0, we calculated the average nonactive HR measures for each participant over the study period.

The Apple Watch uses a built-in accelerometer to track users’ wrist motion and estimate step counts. We first excluded any person-days on which the watch-wearing time was less than 5 hours or daily step counts were less than 1000 [28,29]. Subsequently, we derived average daily step counts for all participants over the study period.

### Covariates

Demographic variables were assessed during examination 3 at the research center. BMI was determined by dividing the participant’s body weight (in kilograms) by the square of their height (in meters). The use of medication was identified based on self-reported information regarding receiving such treatment in the year preceding examination 3. Heart rate–lowering medications were identified using the 2018 American College of Cardiology/American Heart Association/Heart Rhythm Society guideline on the evaluation and the management of patients with bradycardia and included β-blocking agents, calcium channel blocking agents, antiarrhythmic medications, and other medications [30]. Current smoking was defined as individuals who reported smoking at least 1 cigarette per day in the year prior to examination 3. In addition, several laboratory biomarkers, including total cholesterol, high-density lipoprotein, and fasting blood glucose, were measured during examination 3. The presence of diabetes was determined by a fasting blood glucose ≥126 mg/dL, or nonfasting blood glucose ≥200 mg/dL, or the use of any blood glucose–lowering medication. Hypertension was defined as systolic blood pressure (SBP) ≥140 mm Hg, diastolic blood pressure ≥90 mm Hg, or use of any antihypertensive medication. Hyperlipidemia was defined as fasting total cholesterol ≥200 mg/dL, triglyceride ≥150 mg/dL, or use of any lipid-lowering medication. Prevalent CVD was defined as a history of heart failure, myocardial infarction, angina, stroke, intermittent claudication, coronary insufficiency, or transient ischemic attack occurring any time from examination 1 to examination 3 that was reviewed by a panel of senior investigators using all available evidence including hospital records.

### Statistical Analyses

In primary analyses, we examined the associations of CPET fitness measures as independent variables with both average nonactive HR and daily step counts as dependent variables. We selected smartwatch data as the dependent variable because temporally the smartwatch data were collected after the CPET. Peak VO_2_ and VO_2_ at VAT were right skewed and were natural—logarithmically transformed for analysis. The CPET measures were then standardized with a mean of 0, with an SD of 1 prior to analyses. We performed multivariable linear regression models based on different sets of covariates. In model 1, the covariates included age, sex, and self-reported race and ethnicity. In model 2, we additionally adjusted for BMI, current smoking status, total cholesterol, high-density lipoprotein, fasting glucose, diabetes status, resting SBP, prevalent CVD, lipid-lowering treatment status, hypertension treatment status, HR-lowering treatment status (only in analysis of nonactive HR), watch-wearing time (only in the analysis of daily steps), the season of enrollment, and the state of residence.

Furthermore, we conducted a sensitivity analysis to examine the association of CPET measures with nonactive HR with additional adjustment for daily step counts. To investigate whether we had consistent associations between daily steps and CPET fitness measures with a previous study that included 2070 FHS participants and used step data derived from waist-worn accelerometers, we additionally conducted a confirmatory analysis, following their methodology to treat daily step counts as independent variables and CPET fitness measures as dependent variables [7]. Furthermore, to mitigate the impact of insufficient volitional effort, we conducted a sensitivity analysis on a subset of participants who achieved a respiratory exchange ratio (RER) of 1.05 or higher.

For secondary analyses, we evaluated for effect modification by sex, age (older than 53 years or 53 years and younger, the median age in years), and BMI (a 3-level categorical variable: <25, ≥25 but <30, and ≥30 kg/m^2^) on the associations of smartwatch variables with CPET measures using multiplicative interaction terms, as well as stratified analyses by these clinical variables.

In addition to the 662 participants who met the inclusion criteria for analyses of both nonactive HR and daily step data, 215 participants who were excluded from analyses of nonactive HR data met the inclusion criteria for analyses of daily step data. Hence, as a sensitivity analysis, we investigated the association between CPET fitness measures and daily steps in a larger sample including these 215 participants (N=877; Figure 1).

All analyses were performed in R (version 4.3.0; R Foundation for Statistical Computing). We used false discovery rate (FDR)–adjusted *P* value (Benjamini-Hochberg) <.05 to determine statistical significance.

## Results

### Characteristics of Study Participants

The primary study sample consisted of 662 participants, with 59% (n=391) women, mean age 53 (SD 9) years, and 91% (n=599) White (Table 1). The mean peak VO_2_ was 27 (SD 7) mL/kg/min for men and 22 (SD 6) mL/kg/min for women and was 99% of the predicted values overall. The participants had a median follow-up of 128 (IQR 65-227) days and a median daily smartwatch wear time of 14 (IQR 13-15) hours. Overall, we analyzed 2 million HR values over 365,240 person-days. The mean inferred nonactive HR of participants was 73 (SD 6) bpm. On average, the participants took 7269 (SD 2760) steps per day.

Of the 3117 participants who underwent CPET, 1813 were also enrolled in the eFHS. These 1813 participants, on average, were younger (52 vs 56 years) and a larger proportion were women (n=1020, 56% vs n=793, 49%) than those not in eFHS. They also tended to be in superior cardiovascular health with better CRF (Table S1 in Multimedia Appendix 1). Of the 1813 participants engaged in both CPET and eFHS, we excluded 1151 participants who did not use a smartwatch (n=763) or had insufficient smartwatch data (n=388). Those excluded had, on average, lower % predicted peak VO_2_ and % predicted maximum HR than the final study sample. However, there was no significant difference in demographics or metabolic characteristics, such as age, sex, and CVD risk factors (Table S2 in Multimedia Appendix 1).

**Table 1:** Participant characteristics.

Characteristics	Overall (N=662)	Men (n=271)	Women (n=391)
Age (years), mean (SD)	53 (9)	54 (9)	52 (9)
White, n (%)	599 (91)	240 (89)	359 (92)
Hyperlipidemia, n (%)	388 (59)	171 (63.1)	217 (55.5)
Diabetes, n (%)	35 (5)	21 (7.7)	14 (3.6)
Hypertension, n (%)	164 (25)	92 (33.9)	72 (18.4)
Prevalent CVD^a^, n (%)	20 (3)	13 (4.8)	7 (1.8)
BMI, mean (SD)	28.0 (5.3)	29.4 (5.0)	27.1 (5.3)
Peak VO_2_^b^ (mL/kg/min), mean (SD)	23.7 (6.9)	26.6 (6.8)	21.6 (6.1)
% Predicted peak VO_2_, mean (SD)	99.0 (20.8)	96.6 (18.7)	100.6 (22.1)
VO_2_ at VAT^c^ (mL/kg/min), mean (SD)	12.7 (3.7)	13.9 (3.8)	12.0 (3.4)
V_E_/VCO_2_^d^, mean (SD)	27.0 (2.7)	26.3 (2.5)	27.4 (2.7)
% Predicted maximum HR^e^, mean (SD)	90.4 (9.7)	90.4 (10.3)	90.5 (9.2)
SBP/W^f^ slope (mm Hg/W), mean (SD)	0.34 (0.14)	0.29 (0.1)	0.38 (0.15)
Follow-up days, median (IQR)	128 (65-227)	144 (70-246)	113 (63-205)
Number of HR records, median (IQR)	1836 (889-3559)	2062 (960-4063)	1632 (777-3203)
Watch wear time (hour/day), median (IQR)	14 (13-15)	14 (13-15)	14 (13-15)
Nonactive HR (bpm), mean (SD)	73 (6)	73 (7)	73 (6)
Daily steps, mean (SD)	7269 (2760)	7226 (2866)	7299 (2688)

^a^CVD: cardiovascular disease.

^b^VO_2_: oxygen uptake.

^c^VAT: ventilatory anaerobic threshold.

^d^V_E_/VCO_2_: ventilatory efficiency.

^e^HR: heart rate.

^f^SBP/W: systolic blood pressure-to-workload.

### Association of Cardiorespiratory Fitness With Nonactive HR

**Figure 2:**
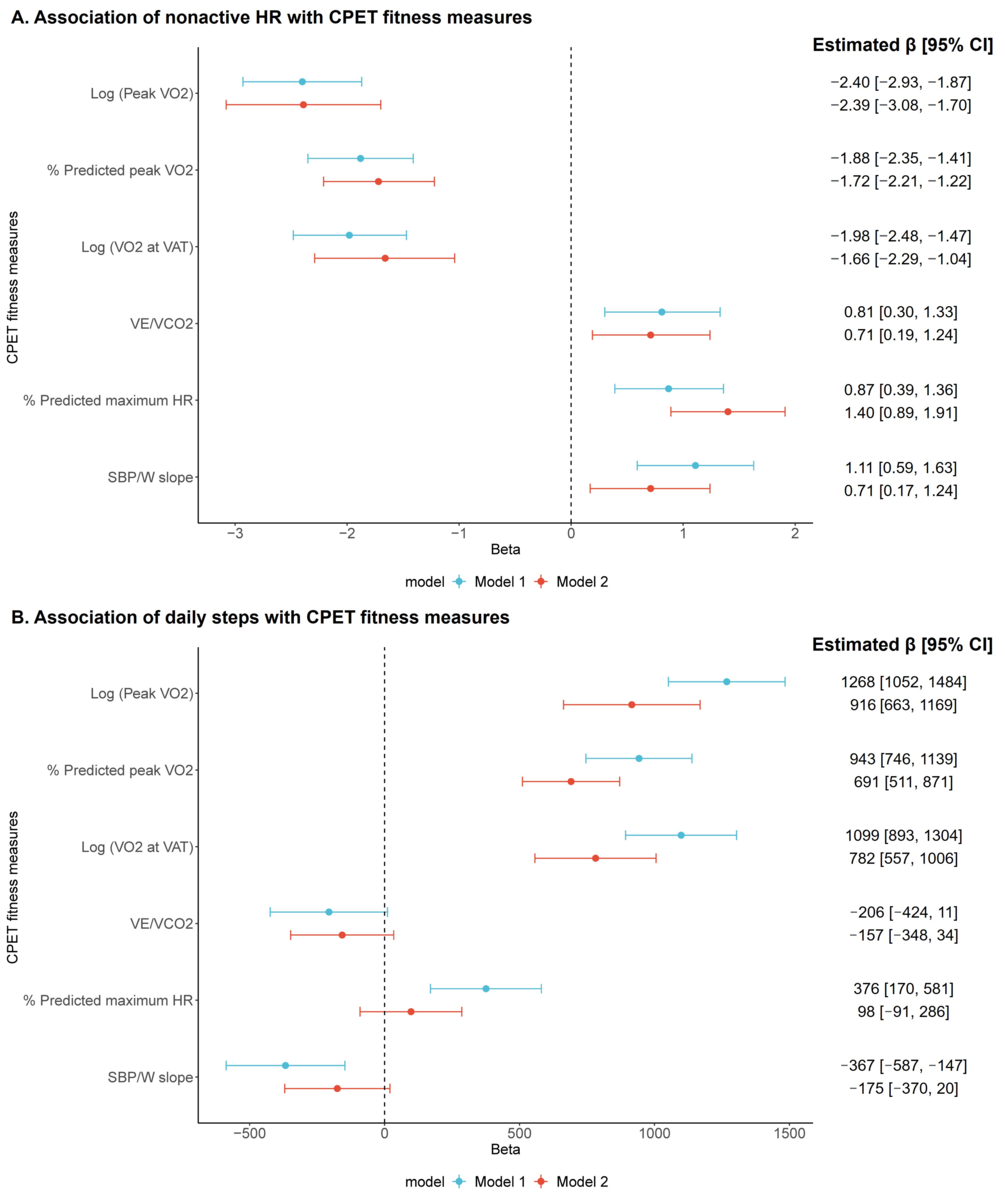
Forest plots of associations between smartwatch-based measures and CPET fitness measures. CPET measures were standardized with a mean of 0 and SD of 1 prior to analysis. Peak VO_2_ and VO_2_ at VAT were standardized after natural log-transformation. % Predicted peak VO2 was calculated using the Wasserman equation. The predicted maximum HR was calculated using the Tanaka formula. (A) Nonactive HR. (B) Daily steps. CPET: cardiopulmonary exercise testing; HR: heart rate; SBP/W: systolic blood pressure-to-workload; VAT, ventilatory anaerobic threshold; VE/VCO_2_: ventilatory efficiency; VO_2_: oxygen uptake.

Adjusting for age, sex, and self-reported race and ethnicity, we observed that each 1-SD higher (favorable) value in log_e_ peak VO_2_ (estimated β=2.4; FDR*-*adjusted *P*<.001), % predicted peak VO_2_ (estimated β=1.88; FDR-adjusted *P*<.001), and log_e_ VO_2_ at VAT (estimated β=1.98; FDR-adjusted *P*<.001) was associated with a lower nonactive HR (Table 2 and Figure 2A). Results also showed that a higher nonactive HR was associated with higher (unfavorable) value in V_E_/VCO_2_ (estimated β=.81; FDR-adjusted *P*=.002) and SBP/W slope (estimated β=1.11; FDR-adjusted *P*<.001), as well as higher % predicted maximum HR (estimated β=.87; FDR*-*adjusted *P*<.001). In the multivariable-adjusted model (model 2), we observed slight attenuation of the effect estimates, which remained statistically significant. Adjusted *R*^2^ of model 2 ranged from 0.07 to 0.12 (Table S3 in Multimedia Appendix 1). Additional adjustment for smartwatch-based daily steps in sensitivity analyses attenuated the association between CPET fitness measures and nonactive HR by around 10%-20% compared with model 2 (Table S4 in Multimedia Appendix 1). The association of CPET fitness measures with nonactive HR persisted after excluding 21 participants with peak RER <1.05 (Table S5 in Multimedia Appendix 1).

**Figure 3:**
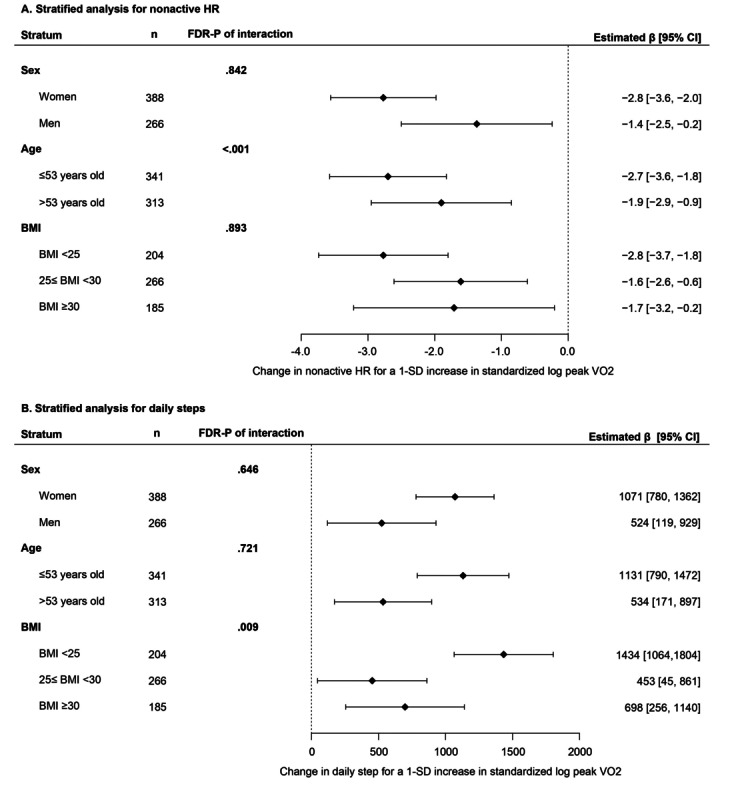
Forest plots of association between smartwatch-based measures and peak VO_2_ stratified by sex, age, and BMI. Peak VO_2_ was standardized with mean of 0 and SD of 1 after natural log-transformation. (A) Nonactive HR. (B) Daily steps. FDR-P: false discovery rate–adjusted *P* values; HR: heart rate; VO_2_: oxygen uptake.

We further assessed whether nonactive HR had different associations with CPET fitness measures between men and women and by age (above and below the median age) and BMI categories. We observed evidence of effect modification by age (FDR-adjusted *P* value of multiplicative interaction <.001; Table S6 in Multimedia Appendix 1) on the association of peak VO_2_ with nonactive HR, such that each 1-SD higher log_e_ peak VO_2_ was associated with a larger decrease in the observed nonactive HR in participants younger than 53 years compared with older participants (53 years and older). In addition, stratified analyses showed that the associations between higher peak VO_2_ (both in mL/kg/min and % predicted) and lower nonactive HR remained significant across strata of sex, age, and BMI category (Figure 3A and Figure S2 in Multimedia Appendix 1). Apart from peak VO_2_, age also modified the association of % predicted peak VO_2_, VO_2_ at VAT, and % predicted maximum HR on nonactive HR (FDR*-*adjusted *P* value of multiplicative interaction <.05). We did not find significant effect modification by sex or BMI of the association between CPET fitness measures and nonactive HR.

**Table 2:** Associations of smartwatch-based measures with CPET^a^ fitness measures.^b^

CPET measure^c^	Nonactive HR^d^ (dependent variable)						Daily steps (dependent variable)					
	Model 1 (n=662)			Model 2 (n=654)			Model 1 (n=662)			Model 2 (n=654)		
	Estimated β	SE	FDR^e^-adjusted *P* value	Estimated β	SE	FDR-adjusted *P* value	Estimated β	SE	FDR-*P*	Estimated β	SE	FDR-adjusted *P* value
Peak VO_2_^f^	–2.40	0.27	<.001	–2.39	0.35	<.001	1268	110	<.001	916	129	<.001
% Predicted peak VO_2_^g^	–1.88	0.24	<.001	–1.72	0.25	<.001	943	100	<.001	691	92	<.001
VO_2_ at VAT^h^	–1.98	0.26	<.001	–1.66	0.32	<.001	1099	105	<.001	782	115	<.001
V_E_/VCO_2_^i^	0.81	0.26	.002	0.71	0.27	.009	206	111	.06	–157	98	.13
% Predicted maximum HR^j^	0.87	0.25	<.001	1.40	0.26	<.001	376	105	<.001	98	96	.31
SBP/W^k^ slope	1.11	0.26	<.001	0.71	0.27	.01	367	112	.001	–175	99	.12

^a^CPET: cardiopulmonary exercise testing.

^b^Model 1 included age, sex, and self-reported race and ethnicity as covariates. Model 2 was additionally adjusted for body mass index, smoking status, total cholesterol, high-density lipoprotein, fasting glucose, diabetes status, resting systolic blood pressure, prevalent CVD, lipid-lowering treatment status, hypertension treatment status, HR-lowering treatment status (only in analysis of nonactive HR), watch-wearing time (only in analysis of daily steps), season of enrollment, and state of residence.

^c^CPET measures were standardized with a mean of 0 and an SD of 1 prior to analysis; peak VO_2_ and VO_2_ at VAT were standardized after natural log transformation.

^d^HR: heart rate.

^e^FDR: false discovery rate

^f^VO_2_: oxygen uptake.

^g^% Predicted peak VO_2_ was calculated using the Wasserman equation.

^h^VAT: ventilatory anaerobic threshold.

^i^V_E_/VCO_2_: ventilatory efficiency.

^j^Predicted maximum HR was calculated using the Tanaka formula.

^k^SBP/W: systolic blood pressure-to-workload.

### Association of Cardiorespiratory Fitness With Daily Steps

Next, we evaluated the relationship between CPET fitness measures and smartwatch-based daily steps. Greater daily steps were significantly related to a higher peak VO_2_, % predicted peak VO_2_, and VO_2_ at VAT for both model 1 and model 2 (FDR-adjusted *P*<.001; Table 2 and Figure 2B). Each 1-SD higher log_e_ peak VO_2_ was associated with 1268 more daily steps in model 1 and 916 more daily steps after adjusting for additional covariates in model 2. We observed no association of V_E_/VCO_2_ with daily steps although the directionality of β estimates was as expected. Both % predicted maximum HR (estimated β=376; FDR-adjusted *P*<.001) and SBP/W slope (estimated β=367; FDR-adjusted *P*=.001) were significantly associated with daily steps in model 1, whereas in model 2, the associations were attenuated (FDR-adjusted *P*>.05). Model 2 adjusted *R*^2^ ranged from 0.3 to 0.36 (Table S3 in Multimedia Appendix 1). A sensitivity analysis restricted to participants with peak RER ≥1.05 (n=641) yielded concordant results (Table S5 in Multimedia Appendix 1). Furthermore, we observed consistent effect sizes compared with a previous study [7] in a confirmatory analysis for which CPET fitness measures were considered as the dependent variable and daily steps (measured by accelerometers worn for a maximum of 8 days) were the independent variable (Table S7 in Multimedia Appendix 1).

Moreover, we observed significant effect modification by BMI on the associations of daily steps with peak VO_2_, % predicted peak VO_2_, and VO_2_ at VAT (FDR-adjusted *P* value of multiplicative interaction <.05; Table S8 in Multimedia Appendix 1) for which the effect sizes were significantly greater in participants with BMI <25 kg/m^2^ compared to higher BMI categories. The effect of % predicted maximum HR was also modified by BMI (FDR-adjusted *P* value of multiplicative interaction=.048), but the greatest effect size was observed among participants with BMI ≥30. Stratified analyses demonstrated that higher peak VO_2_ (both in mL/kg/min and % predicted) was significantly associated with more daily steps across strata of sex, age, and BMI category (Figure 3B; Figure S2 in Multimedia Appendix 1). The association of CPET fitness measures on daily steps was largely consistent across sex and age categories (FDR-adjusted *P* value of multiplicative interaction >.05).

In a sensitivity analysis, we examined the association of CPET fitness measures with daily steps using the larger sample (n=877) with 215 additional participants (Figure 1). The results for peak VO_2_, % predicted peak VO_2_, VO_2_ at VAT, % predicted maximum HR, and SBP/W slope remained very similar to the primary analysis with a sample size of 662 participants (Table S9 in Multimedia Appendix 1). Nevertheless, we observed that higher V_E_/VCO_2_ was significantly associated with lower daily steps in the larger sample (FDR-adjusted *P*<.05), which was not revealed by the primary analysis.

## Discussion

### Principal Findings

In our community-based sample of middle age and older adults, we observed an association between higher peak VO_2_ and lower nonactive HR measured from a consumer-grade smartwatch, with every 1.3 mL/kg/min higher peak VO_2_ corresponding to a 2.4-bpm lower nonactive HR. The association persisted after adjusting for CVD risk factors, prevalent CVD, and background physical activity. In addition to peak VO_2_ (an assessment of global CRF), lower nonactive HR was associated with complementary exercise response patterns such as higher pre-VAT VO_2_, better ventilatory efficiency, and favorable blood pressure response to exercise. Our study also observed an association between higher peak VO_2_ and physical activity with 1.3 mL/kg/min higher peak VO_2_ associated with nearly 1000 more daily steps. While associations between peak VO_2_ and daily steps were observed across strata of age, sex, and BMI, the associations were greater in individuals with normal BMI. Using wearable devices rather than research-grade accelerometers provides the advantage of direct feedback to the participants as well as the ability to incorporate information over a longer time horizon. These findings suggest that HR and daily steps measured from wearable devices may provide one approach to empower and motivate individuals to monitor physiologic measures to improve CRF and overall cardiovascular health.

Our findings are consistent with the UK Fenland Study, a community-based study of adults that noted strong cross-sectional and longitudinal relationships between resting HR and CRF [31]. In that study, resting HR was inversely related to CRF, and within-person change in HR was associated with within-person change in CRF. Furthermore, during the COVID-19 pandemic, a subsample of adults in the UK Fenland Study used a smartphone app for remote HR monitoring and investigators observed that resting HR trajectories differed by prepandemic CRF level. Others have also observed an inverse association between resting HR and CRF [32,33] and importantly between resting HR and adverse outcomes such as mortality independent of physical fitness [32,34]. Our work extends these studies by examining the association of CPET fitness measures with measures of HR obtained remotely from a smartwatch continuously over longer periods of time. Technology now permits remote HR monitoring at the individual and population levels providing a way to track fitness over time and may provide researchers with a population-level biomarker of CRF [31]. Our findings on the association of daily steps with CRF are consistent with a previous study involving 2070 FHS participants and using waist-worn accelerometry step data [7].

A key feature of our study is that we used multiparametric data from a consumer-grade smartwatch to identify HR values measured during nonactive periods. This approach has several potential strengths. First, it derives a meaningful metric of HR regulation from extant smartwatch data rather than requiring special acquisition steps. Second, nonactive HR encompasses many “resting” activities of daily life (eg, working and watching TV) that may lead to differential HR effects when compared with resting HR assessed in a clinical setting. Third, consumer-grade smartwatches conveniently gather a substantial amount of data. In this study, we analyzed 2 million HR values over 365,240 person-days among 662 participants. While it is established that an exaggerated HR increase at the onset of exercise may be a sign of cardiovascular risk [35], whether a lower HR during normal daily nonactive activities is associated with better health outcomes has not been previously evaluated to our knowledge. Our findings suggest that individuals with higher CRF also have lower HR with nonactive daily activities. The observation of effect modification of this association by age such that older individuals demonstrate an attenuated association of CRF and nonactive HR is also intriguing. This finding may be partially attributable to additional comorbidities that may affect HR control (eg, autonomic function) in older individuals and suggests that nonactive HR may be a better correlate of CRF in younger individuals. Finally, not only was global CRF associated with lower nonactive HR but so were lower (better) ventilatory efficiency and BP response to exercise. These observations may suggest that lower nonactive HR reflects multiple components of CRF.

People spend very little time engaged with a physician or other health care provider but spend an estimated 5000 hours each year in activities that can influence their health [36]. National survey data reveal that nearly half of all US adults report using wearable devices daily, and the majority are willing to share their health data with health care providers [37]. A substantial majority of Americans across a range of demographic groups own a smartphone [9] and nearly 90% (n=1800) of users look at their phone within 1 hour of waking and throughout the day [38], providing opportunities to engage adults with monitoring HR, daily steps, and other health metrics in both urban and rural locations where health care access may be challenging. Moreover, many large research programs such as the *All of Us* research program are using wearables to gather HR and physical activity data with the ability to link the data to health outcomes available in electronic health records [39,40]. By demonstrating relations of CRF to 2 measures that are relatively straightforward to assess via wearable devices, this investigation provides potentially useful metrics to monitor with the goals of maintaining or improving CRF.

Another strength of this study is that the eFHS is embedded in the FHS, a long-running observational cohort study with deeply characterized participants with directly measured CVD risk factors and validated CVD outcomes. This allowed us to account for risk factors and CVD in our association analyses.

### Limitations

Our study also has limitations. The eFHS participants were primarily White, well-educated, and healthier than other FHS participants and thus our results may not be generalizable to more diverse samples, other sociodemographic groups, or geographic regions. Our study smartwatch was the Apple Watch version 0. While the Apple Watch has shown good accuracy for HR [41], we did not have motion context information (ie, sedentary or active) for most HR returns. Based on HR return intervals and hourly step counts, we used an imputation method to identify nonactive HR, which was highly correlated with known sedentary HR (Figure S3 in Multimedia Appendix 1). Others have used similar algorithms to identify resting HR from smartwatches or Fitbit data [42]. Our nonactive HR (mean 73, SD 6 bpm) was lower than “known resting HR” (geometric mean 81.8, SD 19.6 bpm) measured at any time by an app that uses a smartphone camera in the Health eHeart cohort [42]. More than 30% (n=2414) of their sample were younger than 30 years, which might explain the higher HR. In contrast, our nonactive HR was higher than resting HR (59-66 bpm) measured in the Michigan Predictive Activity and Clinical Trajectories in Health study [43]. Some of the differences could be due to differences in study samples as HR was observed to differ by demographics (eg, age, sex, race, ethnicity, and BMI).

### Conclusions

This study demonstrated that lower nonactive HR and higher daily steps were each associated with better CRF measures. Our findings suggest the potential use of consumer-grade smartwatches in capturing key physiological metrics that correlate with cardiovascular health and CRF. We demonstrate that peak VO_2_, an established marker of CRF, is associated with nonactive HR and daily steps measured via smartwatches in a community-based sample of middle-aged and older adults. The associations are robust and persist even after rigorous adjustments and excluding participants who did not expend adequate volitional effort. The consistency of our results with previous studies further supports the credibility of our findings. Our research reveals the advantage of leveraging smartwatches in modern health monitoring. These devices not only offer a convenient method for long-term data capture in real-world environments but also provide direct feedback, providing the potential to motivate users to engage in health management. The smartwatch-based physiological metrics, such as nonactive HR and daily steps, may be a reliable proxy for global CRF as our study suggests. However, the limited generalizability of our findings underscores the necessity for further research in more diverse and representative populations. By understanding the correlations between physiological metrics captured by wearable devices and established health indicators, we can potentially leverage smartwatch-based physiological metrics as a population-level biomarker of fitness in epidemiological settings.

## Appendix

Multimedia Appendix 1 Additional figures and tables supporting the analysis presented in the main text.

## Abbreviations

CPET: cardiopulmonary exercise testing
CRF: cardiorespiratory fitness
CVD: cardiovascular disease
eFHS: electronic Framingham Heart Study
FDR: false discovery rate
FHS: Framingham Heart Study
HR: heart rate
RER: respiratory exchange ratio
SBP: systolic blood pressure
SBP/W slope: systolic blood pressure-to-workload slope
VAT: ventilatory anaerobic threshold

## Data Availability

The data sets generated and analyzed during this study are available from the corresponding author on reasonable request and will be available at public repositories (DbGAP and BioLINNC).
