# Supplementary material for: Association of Smartwatch-Based Heart Rate and Physical Activity With Cardiorespiratory Fitness Measures in the Community: Cohort Study
Source: J Med Internet Res. 2024 Jun 13;26:e56676. doi: 10.2196/56676 (PMC11216017; doi:10.2196/56676)
Supplement: Multimedia Appendix 1 [file jmir_v26i1e56676_app1.docx]

**Figure S1.** Distribution of recording interval by motion context in HR data.

**
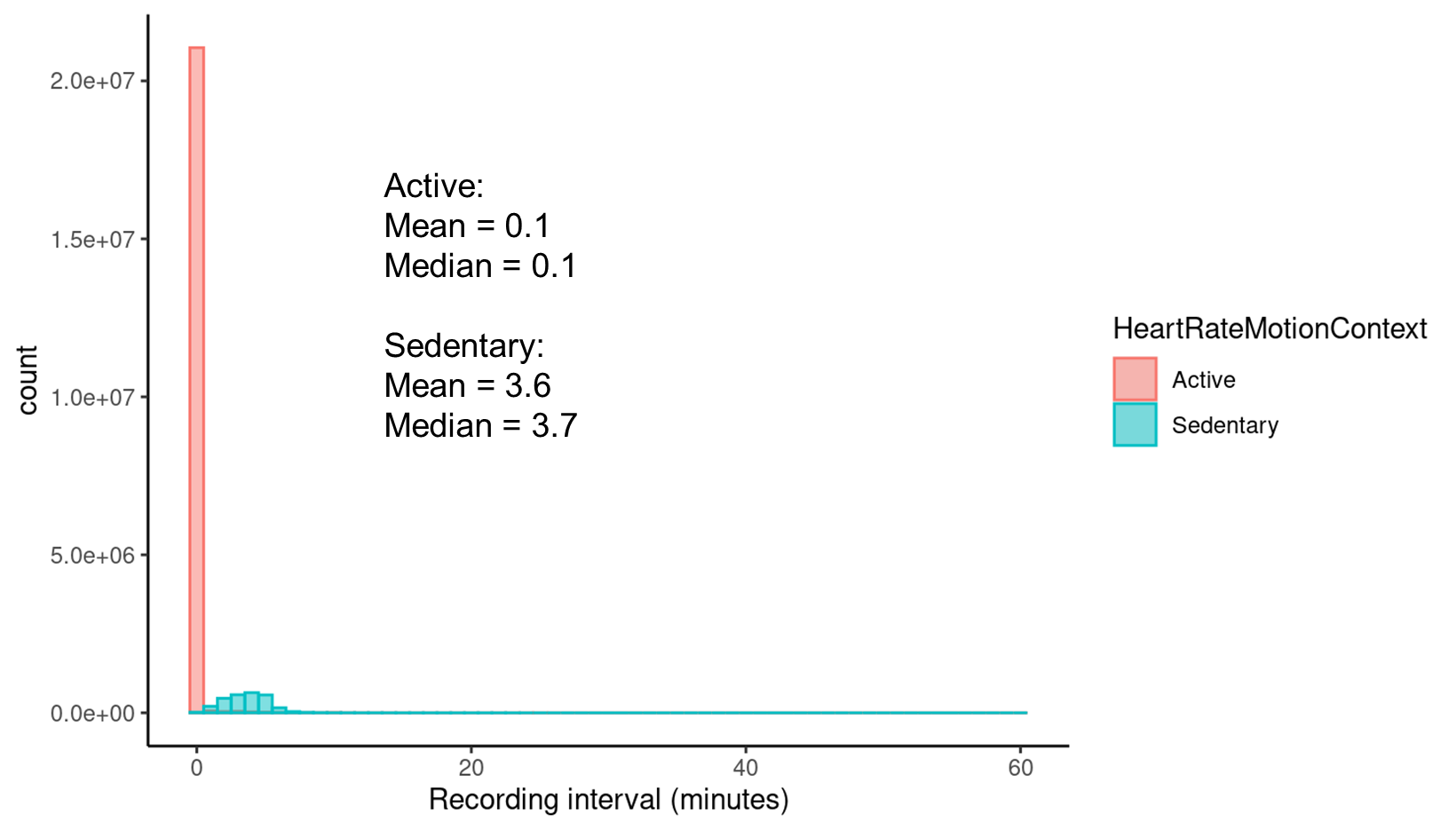
**

**Figure S2.** Association of smartwatch-based measures with peak VO_2_ stratified by sex, age, and BMI.

**
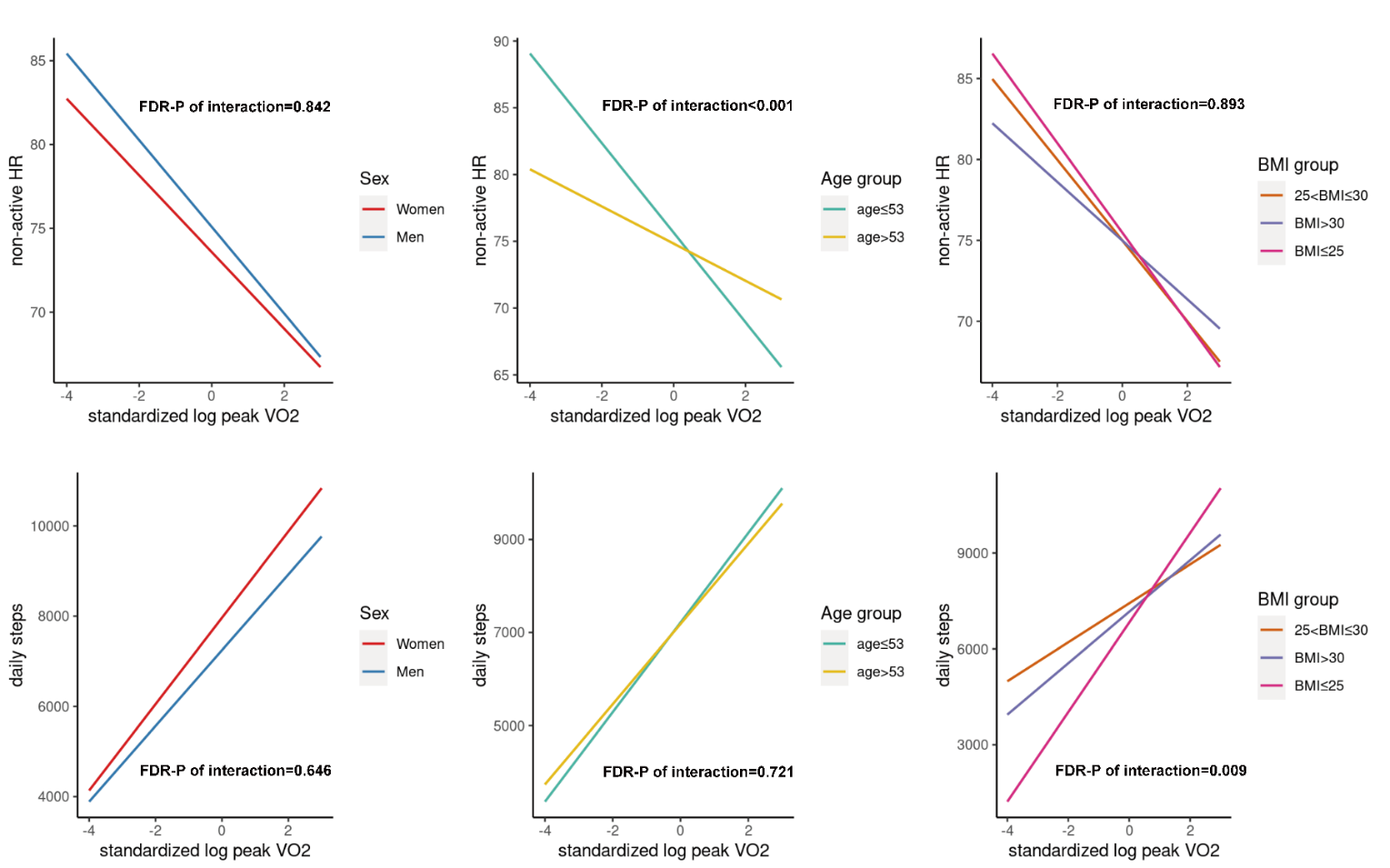
**

**Figure S3.** Correlation between imputed nonactive HR and sedentary HR with known motion context (n=102).

**
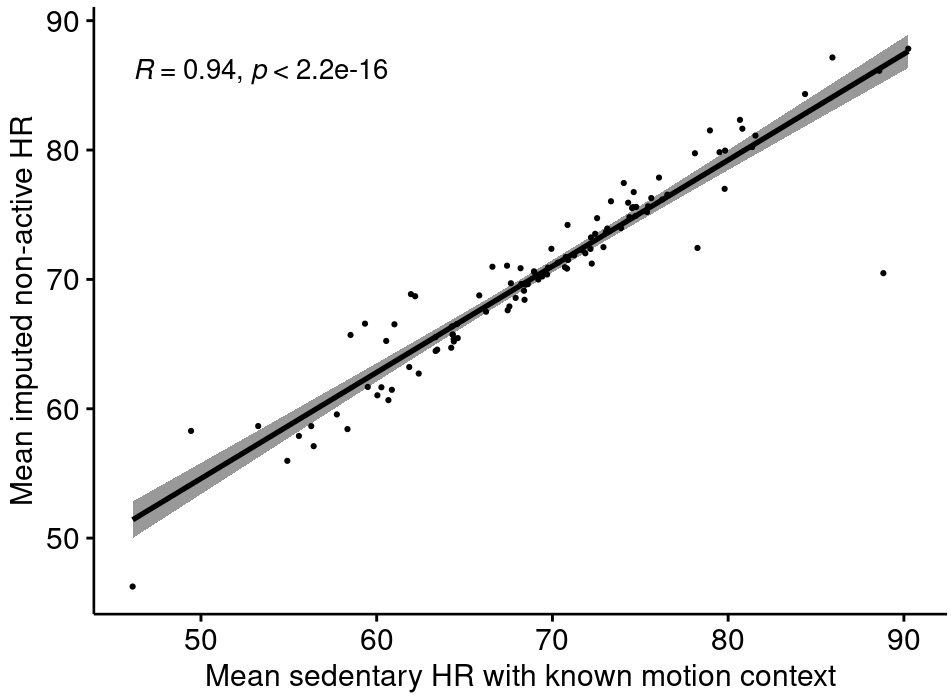
**

**Table S1.** Characteristics of 3117 participants attending CPET.

| **Characteristics** | **Enrolled in CPET but not in eFHS (n=1304)** | **Enrolled in both CPET and eFHS (n=1813)** | ***P* value** |
| --- | --- | --- | --- |
| Age, mean (SD) | 56 (9) | 52 (9) | <.001 |
| Women, n (%) | 642 (49%) | 1020 (56%) | <.001 |
| White, n (%) | 1174 (90%) | 1688 (93%) | .002 |
| Hyperlipidemia, n (%) | 819 (63%) | 1045 (58%) | .004 |
| Diabetes, n (%) | 134 (10%) | 106 (6%) | <.001 |
| Hypertension, n (%) | 427 (33%) | 451 (25%) | <.001 |
| Prevalent CVD^a^, n (%) | 45 (4%) | 53 (3%) | .47 |
| BMI^b^, mean (SD) | 28.6 (5.7) | 28.0 (5.4) | .006 |
| Peak VO_2_^c^, mean (SD), mL/kg/min | 21.4 (6.7) | 23.6 (7.1) | <.001 |
| % Predicted peak VO_2_, mean (SD), % | 90.4 (19.9) | 97.1 (20.4) | <.001 |
| VO_2_ at VAT^d^, mean (SD), mL/kg/min | 12.0 (3.3) | 12.8 (3.8) | <.001 |
| V_E_/VCO_2_^e^, mean (SD) | 27.5 (3.0) | 26.9 (2.7) | <.001 |
| % Predicted maximum HR, mean (SD), % | 86.8 (11.1) | 89.4 (10.1) | <.001 |
| SBP/W slope, mean (SD), mmHg/watts | 0.36 (0.16) | 0.35 (0.14) | .02 |
| Values are depicted n (%); Mean (SD).  ^a^CVD: cardiovascular disease. ^b^BMI: body mass index. ^c^VO_2_: oxygen uptake. ^d^VAT: ventilatory anaerobic threshold. ^e^V_E_/VCO_2_: ventilatory efficiency. | | | |

**Table S2.** Characteristics of 1813 participants enrolled in both CPET and eFHS.

| **Characteristics** | **Final study sample (n=662)** | **Enrolled in eFHS but had none/insufficient watch data (n=1151)** | ***P* value** |
| --- | --- | --- | --- |
| Age, mean (SD) | 53 (9) | 52 (8) | .22 |
| Women, n (%) | 391 (59%) | 629 (55%) | .08 |
| White, n (%) | 599 (91%) | 1089 (95%) | .02 |
| Hyperlipidemia, n (%) | 388 (59%) | 657 (57%) | .63 |
| Diabetes, n (%) | 35 (5%) | 71 (6%) | .49 |
| Hypertension, n (%) | 164 (25%) | 287 (25%) | .98 |
| Prevalent CVD^a^, n (%) | 20 (3%) | 33 (3%) | .97 |
| BMI^b^, mean (SD) | 28.0 (5.3) | 28.0 (5.4) | .85 |
| Peak VO_2_^c^, mean (SD), mL/kg/min | 23.7 (6.9) | 23.6 (7.2) | .92 |
| % Predicted peak VO_2_, mean (SD), % | 99.0 (20.8) | 96.0 (20.1) | .003 |
| VO_2_ at VAT^d^, mean (SD), mL/kg/min | 12.7 (3.7) | 12.9 (3.9) | .46 |
| V_E_/VCO_2_^e^, mean (SD) | 27.0 (2.7) | 26.8 (2.7) | .38 |
| % Predicted maximum HR, mean (SD), % | 90.4 (9.7) | 88.8 (10.3) | .001 |
| SBP/W slope, mean (SD), mmHg/watts | 0.34 (0.14) | 0.35 (0.14) | .59 |
| Values are depicted n (%); Mean (SD).  ^a^CVD: cardiovascular disease. ^b^BMI: body mass index. ^c^VO_2_: oxygen uptake. ^d^VAT: ventilatory anaerobic threshold. ^e^V_E_/VCO_2_: ventilatory efficiency. | | | |

**Table S3.** Adjusted R-squares in primary analyses.

| **CPET measure** | **Non-active HR (Dependent variable)** | | **Daily steps (Dependent variable)** | |
| --- | --- | --- | --- | --- |
|  | **Model 1 (n=662)** | **Model 2 (n=654)** | **Model 1 (n=662)** | **Model 2 (n=654)** |
|  | **Adjusted R^2^** | **Adjusted R^2^** | **Adjusted R^2^** | **Adjusted R^2^** |
| Peak VO_2_ | 0.11 | 0.12 | 0.16 | 0.35 |
| % Predicted peak VO_2_ | 0.09 | 0.12 | 0.11 | 0.36 |
| VO_2_ at VAT | 0.09 | 0.1 | 0.14 | 0.35 |
| V_E_/VCO_2_ | 0.02 | 0.07 | 0.01 | 0.3 |
| % Predicted maximum HR | 0.02 | 0.1 | 0.01 | 0.3 |
| SBP/W slope | 0.03 | 0.07 | 0.01 | 0.3 |

Model 1 included age, sex, and self-reported race and ethnicity as covariates. Model 2 was additionally adjusted for body mass index, smoking status, total cholesterol, high-density lipoprotein, fasting glucose, diabetes status, resting systolic blood pressure, prevalent CVD, lipid lowering treatment status, hypertension treatment status, HR lowering treatment status (only in analysis of non-active HR), watch wearing time (only in analysis of daily steps), season of enrollment, and state of residence.

**Table S4.** Associations of smartwatch-based nonactive HR with CPET fitness measures with additional adjustment for daily step counts.

| **CPET measure*** | **Non-active HR (Dependent variable)** | | |
| --- | --- | --- | --- |
|  | **Model 3 (n=654)** | | |
|  | **Est.beta** | **SE** | ***FDR-P*** |
| Peak VO_2_ | -1.99 | 0.37 | <.001 |
| % Predicted peak VO_2_^§^ | -1.43 | 0.26 | <.001 |
| VO_2_ at VAT | -1.29 | 0.33 | <.001 |
| V_E_/VCO_2_ | 0.65 | 0.26 | .02 |
| % Predicted maximum HR^$^ | 1.50 | 0.25 | <.001 |
| SBP/W slope | 0.61 | 0.27 | .02 |

*CPET measures were standardized with mean of 0 and standard deviation of 1 prior to analysis; Peak VO_2_ and VO_2_ at VAT were standardized after natural log-transformation.

^§^% Predicted peak VO_2_ was calculated using the Wasserman equation.

^$^Predicted maximum HR was calculated using the Tanaka formula.

Model 3 was adjusted for smartwatch-based daily steps in addition to covariates of Model 2.

**Table S5.** Associations of smartwatch-based measures with CPET fitness measures after excluding participants with peak RER <1.05 (n=21).

| **CPET measure*** | **Non-active HR (Dependent variable)** | | | | | | **Daily steps (Dependent variable)** | | | | | |
| --- | --- | --- | --- | --- | --- | --- | --- | --- | --- | --- | --- | --- |
|  | **Model 1 (n=641)** | | | **Model 2 (n=633)** | | | **Model 1 (n=641)** | | | **Model 2 (n=633)** | | |
|  | **Est.beta** | **SE** | ***FDR-P*** | **Est.beta** | **SE** | ***FDR-P*** | **Est.beta** | **SE** | ***FDR-P*** | **Est.beta** | **SE** | ***FDR-P*** |
| Peak VO_2_ | -2.56 | 0.27 | <.001 | -2.66 | 0.36 | <.001 | 1286 | 113 | <.001 | 1003 | 131 | <.001 |
| % Predicted peak VO_2_^§^ | -1.98 | 0.24 | <.001 | -1.88 | 0.26 | <.001 | 959 | 103 | <.001 | 744 | 94 | <.001 |
| VO_2_ at VAT | -2.03 | 0.26 | <.001 | -1.75 | 0.32 | <.001 | 1082 | 107 | <.001 | 781 | 116 | <.001 |
| V_E_/VCO_2_ | 0.73 | 0.26 | .006 | 0.63 | 0.27 | .02 | -212 | 113 | .06 | -161 | 100 | .13 |
| % Predicted maximum HR^$^ | 0.81 | 0.25 | .001 | 1.33 | 0.27 | <.001 | 373 | 107 | <.001 | 147 | 99 | .14 |
| SBP/W slope | 1.15 | 0.27 | <.001 | 0.73 | 0.28 | .01 | -388 | 116 | <.001 | -184 | 103 | .11 |

*CPET measures were standardized with mean of 0 and standard deviation of 1 prior to analysis; Peak VO_2_ and VO_2_ at VAT were standardized after natural log-transformation.

^§^% Predicted peak VO_2_ was calculated using the Wasserman equation.

^$^Predicted maximum HR was calculated using the Tanaka formula.

Model 1 included age, sex, and self-reported race and ethnicity as covariates. Model 2 was additionally adjusted for body mass index, smoking status, total cholesterol, high-density lipoprotein, fasting glucose, diabetes status, resting systolic blood pressure, prevalent CVD, lipid lowering treatment status, hypertension treatment status, HR lowering treatment status (only in analysis of non-active HR), watch wearing time (only in analysis of daily steps), season of enrollment, and state of residence.

**Table S6.** Associations of smartwatch-based nonactive HR with CPET fitness measures stratified by sex, age, and BMI.

| **CPET measure** | **Non-active HR (Dependent variable)** | | | | | |
| --- | --- | --- | --- | --- | --- | --- |
|  | **Stratum** | **n** | **Est.beta** | **SE** | ***FDR-P*** | ***FDR-P* of multiplicative interaction** |
| Peak VO_2_ | Women | 388 | -2.77 | 0.40 | <.001 | .84 |
|  | Men | 266 | -1.37 | 0.58 | .04 |  |
|  | ≤ 53 years | 341 | -2.70 | 0.45 | <.001 | <.001 |
|  | > 53 years | 313 | -1.90 | 0.53 | .001 |  |
|  | BMI < 25 kg/m^2^ | 204 | -2.77 | 0.49 | <.001 | .89 |
|  | 25 ≤ BMI < 30 kg/m^2^ | 266 | -1.61 | 0.51 | .003 |  |
|  | BMI ≥ 30 kg/m^2^ | 185 | -1.71 | 0.77 | .06 |  |
| % Predicted peak VO_2_ | Women | 388 | -2.05 | 0.31 | <.001 | .84 |
|  | Men | 266 | -1.19 | 0.44 | .02 |  |
|  | ≤ 53 years | 341 | -2.05 | 0.32 | <.001 | .04 |
|  | > 53 years | 313 | -1.37 | 0.40 | .001 |  |
|  | BMI < 25 kg/m^2^ | 204 | -2.31 | 0.41 | <.001 | .89 |
|  | 25 ≤ BMI < 30 kg/m^2^ | 266 | -1.25 | 0.39 | .003 |  |
|  | BMI ≥ 30 kg/m^2^ | 185 | -1.29 | 0.55 | .06 |  |
| VO_2_ at VAT | Women | 388 | -2.09 | 0.39 | <.001 | .84 |
|  | Men | 266 | -1.01 | 0.51 | .07 |  |
|  | ≤ 53 years | 341 | -1.68 | 0.40 | <.001 | .009 |
|  | > 53 years | 313 | -1.72 | 0.50 | .001 |  |
|  | BMI < 25 kg/m^2^ | 204 | -1.74 | 0.47 | <.001 | .89 |
|  | 25 ≤ BMI < 30 kg/m^2^ | 266 | -1.59 | 0.44 | .001 |  |
|  | BMI ≥ 30 kg/m^2^ | 185 | -1.23 | 0.72 | .11 |  |
| V_E_/VCO_2_ | Women | 388 | 0.82 | 0.32 | .01 | .84 |
|  | Men | 266 | 0.21 | 0.49 | .67 |  |
|  | ≤ 53 years | 341 | 0.86 | 0.36 | .03 | .08 |
|  | > 53 years | 313 | 0.23 | 0.40 | .56 |  |
|  | BMI < 25 kg/m^2^ | 204 | 1.13 | 0.44 | .01 | .89 |
|  | 25 ≤ BMI < 30 kg/m^2^ | 266 | 0.09 | 0.42 | .84 |  |
|  | BMI ≥ 30 kg/m^2^ | 185 | 0.99 | 0.56 | .11 |  |
| % Predicted maximum HR | Women | 388 | 1.24 | 0.34 | <.001 | .84 |
|  | Men | 266 | 1.63 | 0.43 | .001 |  |
|  | ≤ 53 years | 341 | 0.75 | 0.36 | .04 | .04 |
|  | > 53 years | 313 | 1.79 | 0.40 | <.001 |  |
|  | BMI < 25 kg/m^2^ | 204 | 1.17 | 0.42 | .008 | .89 |
|  | 25 ≤ BMI < 30 kg/m^2^ | 266 | 1.42 | 0.39 | .001 |  |
|  | BMI ≥ 30 kg/m^2^ | 185 | 1.91 | 0.56 | .005 |  |
| SBP/W slope | Women | 388 | 0.72 | 0.33 | .03 | .84 |
|  | Men | 266 | 0.69 | 0.43 | .13 |  |
|  | ≤ 53 years | 341 | 0.71 | 0.36 | .05 | .06 |
|  | > 53 years | 313 | 0.72 | 0.42 | .10 |  |
|  | BMI < 25 kg/m^2^ | 204 | 0.73 | 0.46 | .12 | .89 |
|  | 25 ≤ BMI < 30 kg/m^2^ | 266 | 0.49 | 0.42 | .30 |  |
|  | BMI ≥ 30 kg/m^2^ | 185 | 0.52 | 0.57 | .36 |  |

Stratified models were adjusted for the covariates of Model 2.

**Table S7.** Associations of smartwatch-based daily steps with CPET fitness measures using the same dependent and independent variables as Nayor et al^#^.

| **CPET measure*** | **Daily steps (Independent variable)** | | |
| --- | --- | --- | --- |
|  | **Est.beta** | **SE** | ***FDR-P*** |
| Peak VO_2_ | 0.22 | 0.03 | <.001 |
| % Predicted peak VO_2_^§^ | 0.32 | 0.04 | <.001 |
| VO_2_ at VAT | 0.24 | 0.03 | <.001 |
| V_E_/VCO_2_ | -0.07 | 0.04 | .13 |
| % Predicted maximum HR^$^ | 0.04 | 0.04 | .31 |
| SBP/W slope | -0.07 | 0.04 | .12 |

*CPET measures and daily steps were standardized with mean of 0 and standard deviation of 1 prior to analysis; Peak VO_2_ and VO_2_ at VAT were standardized after natural log-transformation.

^§^% Predicted peak VO_2_ was calculated using the Wasserman equation.

^$^Predicted maximum HR was calculated using the Tanaka formula.

^#^Nayor M, Chernofsky A, Spartano NL, et al. Physical activity and fitness in the community: the Framingham Heart Study. Eur Heart J. 2021;42(44):4565-4575.

Model was adjusted for age, sex, self-reported race and ethnicity, body mass index, smoking status, total cholesterol, high-density lipoprotein, fasting glucose, diabetes status, resting systolic blood pressure, prevalent CVD, lipid lowering treatment status, hypertension treatment status, watch wearing time, season of enrollment, and state of residence.

**Table S8.** Associations of smartwatch-based daily steps with CPET fitness measures stratified by sex, age, and BMI.

| **CPET measure** | **Daily steps (Dependent variable)** | | | | | |
| --- | --- | --- | --- | --- | --- | --- |
|  | **Stratum** | **n** | **Est.beta** | **SE** | ***FDR-P*** | ***FDR-P* of multiplicative interaction** |
| Peak VO_2_ | Women | 388 | 1071 | 149 | <.001 | .65 |
|  | Men | 266 | 524 | 207 | .03 |  |
|  | ≤ 53 years | 341 | 1131 | 174 | <.001 | .72 |
|  | > 53 years | 313 | 534 | 185 | .008 |  |
|  | BMI < 25 kg/m^2^ | 204 | 1434 | 189 | <.001 | .009 |
|  | 25 ≤ BMI < 30 kg/m^2^ | 266 | 453 | 208 | .06 |  |
|  | BMI ≥ 30 kg/m^2^ | 185 | 698 | 226 | .01 |  |
| % Predicted peak VO_2_ | Women | 388 | 835 | 113 | <.001 | .48 |
|  | Men | 266 | 429 | 158 | .03 |  |
|  | ≤ 53 years | 341 | 868 | 126 | <.001 | .35 |
|  | > 53 years | 313 | 450 | 137 | .006 |  |
|  | BMI < 25 kg/m^2^ | 204 | 1172 | 157 | <.001 | .009 |
|  | 25 ≤ BMI < 30 kg/m^2^ | 266 | 430 | 161 | .02 |  |
|  | BMI ≥ 30 kg/m^2^ | 185 | 477 | 162 | .01 |  |
| VO_2_ at VAT | Women | 388 | 957 | 140 | <.001 | .57 |
|  | Men | 266 | 449 | 184 | .03 |  |
|  | ≤ 53 years | 341 | 903 | 153 | <.001 | .72 |
|  | > 53 years | 313 | 537 | 172 | .006 |  |
|  | BMI < 25 kg/m^2^ | 204 | 1172 | 177 | <.001 | .048 |
|  | 25 ≤ BMI < 30 kg/m^2^ | 266 | 602 | 179 | .005 |  |
|  | BMI ≥ 30 kg/m^2^ | 185 | 383 | 209 | .10 |  |
| V_E_/VCO_2_ | Women | 388 | -293 | 116 | .02 | .48 |
|  | Men | 266 | 18 | 177 | .92 |  |
|  | ≤ 53 years | 341 | -210 | 143 | .17 | .72 |
|  | > 53 years | 313 | 23 | 138 | .87 |  |
|  | BMI < 25 kg/m^2^ | 204 | -383 | 177 | .048 | .62 |
|  | 25 ≤ BMI < 30 kg/m^2^ | 266 | -108 | 172 | .53 |  |
|  | BMI ≥ 30 kg/m^2^ | 185 | -13 | 165 | .94 |  |
| % Predicted maximum HR | Women | 388 | 130 | 125 | .30 | .68 |
|  | Men | 266 | -18 | 158 | .92 |  |
|  | ≤ 53 years | 341 | -65 | 139 | .64 | .40 |
|  | > 53 years | 313 | 229 | 139 | .15 |  |
|  | BMI < 25 kg/m^2^ | 204 | 289 | 169 | .11 | .048 |
|  | 25 ≤ BMI < 30 kg/m^2^ | 266 | -293 | 162 | .09 |  |
|  | BMI ≥ 30 kg/m^2^ | 185 | 325 | 162 | .09 |  |
| SBP/W slope | Women | 388 | -285 | 119 | .02 | .48 |
|  | Men | 266 | 54 | 157 | .92 |  |
|  | ≤ 53 years | 341 | -258 | 140 | .10 | .72 |
|  | > 53 years | 313 | -70 | 142 | .75 |  |
|  | BMI < 25 kg/m^2^ | 204 | -241 | 187 | .20 | .36 |
|  | 25 ≤ BMI < 30 kg/m^2^ | 266 | -326 | 168 | .08 |  |
|  | BMI ≥ 30 kg/m^2^ | 185 | 150 | 165 | .44 |  |

Stratified models were adjusted for the covariates of Model 2.

**Table S9.** Associations of smartwatch-based daily steps with CPET fitness measures in a larger sample.

| **CPET measure*** | **Daily steps (Dependent variable)** | | | | | |
| --- | --- | --- | --- | --- | --- | --- |
|  | **Model 1 (n=877)** | | | **Model 2 (n=865)** | | |
|  | **Est.beta** | **SE** | ***FDR-P*** | **Est.beta** | **SE** | ***FDR-P*** |
| Peak VO_2_ | 1201 | 95 | <.001 | 891 | 113 | <.001 |
| % Predicted peak VO_2_^§^ | 895 | 86 | <.001 | 668 | 81 | <.001 |
| VO_2_ at VAT | 1049 | 90 | <.001 | 775 | 100 | <.001 |
| V_E_/VCO_2_ | -247 | 94 | .009 | -201 | 85 | .03 |
| % Predicted maximum HR^$^ | 250 | 91 | .007 | -31 | 85 | .72 |
| SBP/W slope | -348 | 96 | <.001 | -171 | 86 | .06 |

*CPET measures were standardized with mean of 0 and standard deviation of 1 prior to analysis; Peak VO_2_ and VO_2_ at VAT were standardized after natural log-transformation.

^§^% Predicted peak VO_2_ was calculated using the Wasserman equation.

^$^Predicted maximum HR was calculated using the Tanaka formula.

Model 1 included age, sex, and self-reported race and ethnicity as covariates. Model 2 was additionally adjusted for body mass index, smoking status, total cholesterol, high-density lipoprotein, fasting glucose, diabetes status, resting systolic blood pressure, prevalent CVD, lipid lowering treatment status, hypertension treatment status, watch wearing time, season of enrollment, and state of residence.
